# Naloxone Coprescribing and the Prevention of Opioid Overdoses: Quasi-Experimental Metacognitive Assessment of a Novel Education Initiative

**DOI:** 10.2196/54280

**Published:** 2024-10-28

**Authors:** Michael Enich, Cory Morton, Richard Jermyn

**Affiliations:** 1 Department of Internal Medicine, University of Washington, Seattle, WA, United States; 2 Center for Prevention Science, School of Social Work, Rutgers, The State University of New Jersey, 390 George Street, 5th Floor, New Brunswick, NJ, 08901, United States, 1 8489327500; 3 Rowan-Virtua School of Osteopathic Medicine, Rowan University, Glassboro, NJ, United States

**Keywords:** naloxone, coprescribing, prescription, academic detailing, metacognition, metacognitive evaluation, pharmacotherapy, pharmaceutic, pharmaceutical, education, educational intervention, opioid, opioid overdose, harm reduction

## Abstract

**Background:**

Critical evaluation of naloxone coprescription academic detailing programs has been positive, but little research has focused on how participant thinking changes during academic detailing.

**Objective:**

The dual purposes of this study were to (1) present a metacognitive evaluation of a naloxone coprescription academic detailing intervention and (2) describe the application of a metacognitive evaluation for future medical education interventions.

**Methods:**

Data were obtained from a pre-post knowledge assessment of a web-based, self-paced intervention designed to increase knowledge of clinical and organizational best practices for the coprescription of naloxone. To assess metacognition, items were designed with confidence-weighted true-false scoring. Multiple metacognitive scores were calculated: 3 content knowledge scores and 5 confidence-weighted true-false scores. Statistical analysis examined whether there were significant differences in scores before and after intervention. Analysis of overall content knowledge showed significant improvement at posttest.

**Results:**

There was a significant positive increase in absolute accuracy of participant confidence judgments, confidence in correct probability, and confidence in incorrect probability (all *P* values were <.05). Overall, results suggest an improvement in content knowledge scores after intervention and, metacognitively, suggest that individuals were more confident in their answer choices, regardless of correctness.

**Conclusions:**

Implications include the potential application of metacognitive evaluations to assess nuances in learner performance during academic detailing interventions and as a feedback mechanism to reinforce learning and guide curricular design.

## Introduction

In 2020, of the 91,799 drug-related overdoses in the United States, 75% involved an opioid [1]. Naloxone is an invaluable tool to prevent opioid overdose [2], and coprescription initiatives (or programs to encourage providers to prescribe naloxone to patients receiving opioids) are a common, important intervention to reduce fatal overdoses. This is especially true in primary care settings, since eligible patients who meet the Centers for Disease Control and Prevention coprescription guidelines are underprescribed naloxone to take home [3].

Academic detailing programs are educational outreach approaches used to improve clinician decision-making, and they have consistently shown a positive effect on altering prescriber behavior and clinical decision-making [4]. Health systems have implemented naloxone coprescription academic detailing programs with demonstrated positive effects on the number of providers prescribing and patients receiving naloxone [2,5]. Critical evaluations of such programs have shown acceptability and feasibility of such interventions [3], including positive impact on increasing the number of prescriptions despite hesitancy around the logistics of implementation [6] and increasing the number of prescriptions after brief interventions [7].

Learners in the health professions are important allies for combatting overdose; interventions have been developed for health profession students to be trained in overdose education and naloxone distribution [8]. Results from student-focused overdose education and naloxone distribution interventions indicate increases in average participant knowledge around identifying and responding to suspected overdoses [8]. To extend knowledge on changes in participant thinking during educational interventions, one area to consider is the effect on participant metacognition. Here, metacognition refers to the beliefs, attitudes, and confidence related to influencing a particular cognitive task, colloquially summarized as thinking about thinking. The measurement of participant metacognitive processes is especially important in health education because of the importance of the desired outcomes and the need for a life span approach to learning in the health professions. Improvements in metacognition in health education interventions have been linked to improved content knowledge acquisition, improved clinical reasoning, and decreased avoidable errors [9]. However, assessing metacognition is not often a focus of medical education evaluation, and those who wish to evaluate metacognition are often met with a lack of clarity on how to effectively measure it [10].

The dual purposes of this brief report are to (1) present a metacognitive evaluation of a naloxone coprescription academic detailing intervention for health professions students and practitioners and (2) describe the application of a metacognitive evaluation for future medical education interventions.

## Methods

### Data

Participants completed a self-paced, web-based academic detailing naloxone coprescription intervention implemented by Rowan University School of Osteopathic Medicine (RUSOM). This brief continuing medical education (CME)–eligible course provided a standardized, evidence-based curriculum to train RUSOM–affiliated health care providers, administrators, students, and executives across a variety of health care settings on how to implement and sustain naloxone coprescribing programs. Participants were recruited via email, and the only incentive was providing the CME credit at no cost. Consent was provided by agreeing to a question prompt to continue each survey after reading the informed consent documentation.

Data for this analysis came from a 20-item knowledge assessment based on the Centers for Disease Control and Prevention naloxone coprescription guidelines [11], previously validated overdose knowledge assessment instruments [12], as well as guidance based on best practices in implementation science [13]. It was delivered in a pre-post design, where participants completed the knowledge assessment before accessing the educational intervention and after course completion. The course was designed as a single module to be completed in 1 session. Participants were eligible for CME credits after completion if they achieved a passing score; multiple individuals had more than 1 posttest score as they attempted to meet that minimum score. To avoid a bias in results, knowledge scores that came chronologically first were used as the posttest score in all analyses.

To assess metacognition, items were designed with confidence-weighted true-false (CTF) scoring, which combines traditional true-false questions with each learner’s rated confidence for each item (I am confident this is true; I believe this is true, but I am unsure; I believe this is false, but I am unsure; and I am confident this is false). CTF is a useful and simple means to measure both cognitive and metacognitive achievements [14].

### Study Sample

The sample includes any individual who completed both pre- and posttest assessments for the naloxone coprescription educational intervention between dates April 2020 and July 2021. To access the intervention, participants had to register via a university web application (from which voluntary demographic data were derived) and then log in to their learning management system. While the intervention provided an opportunity for CME credits, any individual was able to register for and take the course, including nonprescribers and students.

### Analysis

Descriptive statistics were calculated for individuals in the study sample. For both the pre- and posttest, 3 content knowledge scores were calculated: the summed CTF score (where confidently incorrect scores equal 0 points and confidently correct scores equal 4 points), the percent correct CTF score (based on maximum of 80), and the binary percent correct score (true/false [T/F]) (number correct regardless of confidence or number of items). In addition, metacognitive scores were calculated using the methods described by Dutke and Barenberg [14] and included absolute accuracy of confidence judgments (AC), bias of confidence judgments (BS), confidence correct probability (CCP), confidence incorrect probability (CIP), and discrimination between correct and incorrect decisions (DIS). AC reflects the overall match between participant confidence and the outcome of their choice. An increase in AC suggests that individuals are better able to gauge both when they are confident in correct answers and unconfident in incorrect answers. BS is similar to AC but gives an indication of the direction and severity of participant ability to correctly asses their level of confidence in an answer. Values close to 0 indicate an exact match between confidence or nonconfidence and correctness or incorrectness, positive values suggest overconfidence (more confident but less correct), and negative values indicate underconfidence (less confident but more correct). However, the BS does not indicate the relative contribution of confidence to correct or incorrect answers, and the CCP and CIP are used to discern the respective probabilities of being confident that the answer is correct (CCP) or confident that the answer is incorrect (CIP). A higher CCP score indicates higher confidence when the answer is correct. A lower CIP score indicates less confidence when the answer is incorrect. A high CCP and low CIP suggests improvement in metacognition. Finally, the DIS is the difference between the CCP and CIP probabilities and is used to indicate how reliably a participant discriminates between correct and incorrect answers, with higher values indicating appropriate participant metacognitive monitoring and the ability to discriminate between concepts that are known and those that need reinforcement [14]. To correct for a left-skewed distribution of assessment values, Wilcoxon signed rank analyses were applied to assess changes in individual scores between pre- and posttest assessments. Finally, Rosenthal correlations were calculated to determine the effect size of the intervention on each metacognitive score. Item-level examinations of CTF distribution were completed to add context to the metacognitive outcomes and identify concepts in the naloxone coprescription framework that may need reinforcement. McNemar tests were used to determine whether there was a significant change in correctness from pre- to posttest for each item. Statistical analyses were completed using Stata 17 (StataCorp LLC).

### Ethical Considerations

This study was approved by the Rutgers University Institutional Review Board (ID2019000275). Participants were provided informed consent at pretest and posttest, and data were deidentified prior to analysis. The course and CME credit were provided at no cost to participants, and no additional compensation was provided.

## Results

Sample descriptive statistics are shown in Table 1; 307 individuals completed both pre- and posttests. As shown in Table 2, analysis of overall test scores showed a statistically significant improvement in content knowledge after completing the educational intervention, both in CTF score and binary correct-incorrect score. For both, the effect size of the intervention was moderate.

Significant differences in metacognitive scores suggest potential improvements in metacognitive monitoring occurred during the intervention. There is a statistically significant increase in absolute AC with a moderate effect size, suggesting that after intervention individuals are better able to gauge when they are *confident* in *correct* answers and *unconfident* in *incorrect* answers. For BS, median response values changed from negative to positive with a strong effect size, suggesting an overall change from being underconfident (negative values) in answer choices to appropriately confident (null or positive values) after intervention. Both CCP and CIP had a significant, positive change after intervention with strong effects. There was a significant decrease in DIS score after intervention with a very low effect size, which likely reflects an underlying increase in confidence in incorrect answers after academic detailing.

Table 3 shows the CTF and binary T/F frequencies for each item and an indication of significant change from pre- to posttest using McNemar test. Most items saw their binary correct answers increase at posttest; only 1 item (item 15) saw a significant decline in correct answers (*t*_306_=−4.41; *P*=.04). This item was part of a conceptual group of questions (items 7, 12, and 15) on determining individual risk of overdose using the Risk Index for Overdose or Serious Opioid-Induced Respiratory Depression (RIOSORD) tool. From a metacognitive perspective, this group of questions also saw the frequency of confident incorrect answers increase at posttest between 117% and 350%.

**Table 1.** Demographic characteristics of participants (N=307).

Characteristics	Participants	
**Sex, n (%)**		
Male	77 (25.1)	
Female	106 (34.5)	
Undisclosed	124 (40.4)	
**Race/ethnicity, n (%)**		
White/non-Hispanic	88 (28.7)	
Black/non-Hispanic	18 (5.9)	
Hispanic	13 (4.2)	
Native American	3 (1.0)	
Asian/Pacific Islander	4 (1.3)	
Undisclosed	181 (59.0)	
**Credentials, n (%)**		
Health professions students	213 (69.4)	
Prescribers (MD^a^, DO^b^, NP-C^c^, or PA^d^)	48 (15.6)	
Pharmacists	3 (1.0)	
Other health professional	4 (1.3)	
Undisclosed	39 (12.7)	
**Age (years), mean (SD)**	32 (11.6)	

a MD: medical doctor.

b DO: doctor of osteopathic medicine.

c NP-C: nurse practitioner.

d PA: physician’s assistant.

**Table 2.** Naloxone coprescription program metacognitive scores (N=307).

	Preintervention			Postintervention			*df*	*z*	Effect size^a^
	Median	IQR	Range	Median	IQR	Range			
CTF^b^ overall score	36	32 to 40	17 to 51	43	33 to 48	18 to 58	306	−9.41^c^	−0.54 (moderate)
Binary true/false score	60	53.3 to 66.7	28.3 to 85	71.7	55 to 80	30 to 97	306	−9.41^c^	−0.54 (moderate)
Absolute accuracy of confidence judgments	0.55	0.45 to 0.65	0.15 to 0.95	0.65	0.55 to 0.80	0.2 to 1	306	−9.42^c^	−0.54 (moderate)
Bias of the confidence judgments	−0.35	−0.50 to −0.10	−0.85 to 0.65	0.10	−0.15 to 0.25	−0.80 to 0.70	306	−13.08^c^	−0.75 (strong)
Confident correct probability	0.36	0.17 to 0.62	0 to 1	0.88	0.64 to 1	0 to 1	306	−13.59^c^	−0.78 (strong)
Confident incorrect probability	0.14	0 to 0.43	0 to 1	0.80	0.38 to 1	0 to 1	306	−12.82^c^	−0.73(strong)
Discrimination between correct and incorrect decisions	0.11	0 to 0.26	−0.63 to 0.92	0	0‐0.21	−0.54 to 1	306	2.85^d^	0.16(weak)

a Rosenthal correlation (1991).

b CTF: confidence-weighted true-false.

c *P*<.001.

d *P*<.01 (for this entry: *P*=.004).

**Table 3.** Item-level frequency distribution of confidence-weighted true-false (CTF) and binary true (T)/false (F) choices at pre- and posttests (N=307).

	Pretest		Posttest		McNemar test on binary pre- and postperformance		Confident incorrect answers, % change
	CTF (%)	Binary choice (%)	CTF (%)	Binary choice (%)	Test statistic	*P* value	
***Item 1: Naloxone coprescription efforts have been shown to increase access to naloxone for high-risk patients only in primary care settings. [Correct: F]***					2.47	.16	100
Sure True	11	True: 38	22	True: 33			
Unsure True	27		11				
Unsure False	36	False: 62	11	False: 66			
Sure False	26		55				
***Item 2: Higher doses of naloxone may be safely used if a person is suspected of overdosing from synthetic opioids such as Fentanyl. [Correct: T]***					26.18	<.001	−28.5
Sure False	7	False: 27	5	False: 11			
Unsure False	19		6				
Unsure True	45	True: 73	22	True: 88			
Sure True	28		66				
***Item 3: Clinicians can prescribe only naloxone to patients receiving opioid prescriptions. [Correct: F]***					1.25	.26	64
Sure True	11	True: 28	18	True: 26			
Unsure True	17		8				
Unsure False	30	False: 72	12	False: 75			
Sure False	42		63				
***Item 4: A person under the influence of an opioid can be arrested and charged for being under the influence of a controlled substance if he or she seeks*** ***medical assistance for himself or herself or someone else. [Correct: F]***					0.91	.34	5
Sure True	6	True: 22	14	True: 21			
Unsure True	16		7				
Unsure False	21	False: 78	5	False: 80			
Sure False	57		75				
***Item 5: The cheapest form of naloxone is the naloxone autoinjector made by Evzio. [Correct: F]***					16.20	<.001	129
Sure True	7	True: 47	18	True: 33			
Unsure True	40		15				
Unsure False	37	False: 53	6	False: 67			
Sure False	16		61				
***Item 6: Writing a prescription for Evzio, Narcan, or generic will each result in a patient receiving the same product. [Correct: F]***					13.23	<.001	82
Sure True	17	True: 56	31	True: 44			
Unsure True	39		13				
Unsure False	29	False: 44	13	False: 55			
Sure False	15		42				
***Item 7: Naloxone should be coprescribed to patients only with a RIOSORD**^a^ **score of >18. [Correct: F]***					0.81	.37	350
Sure True	8	True: 59	36	True: 56			
Unsure True	51		20				
Unsure False	30	False: 41	11	False: 44			
Sure False	11		33				
***Item 8: Facilitators involved in leading the implementation of a naloxone coprescribing program are limited to clinical staff. [Correct: F]***					19.76	<.001	−32
Sure True	9	True: 38	16	True: 26			
Unsure True	30		10				
Unsure False	31	False: 62	14	False: 74			
Sure False	30		60				
***Item 9: Academic detailing is a service provided by academic professionals (ie, faculty at educational institutions) who provide clinicians with information on new clinical guidelines and how to implement them. [Correct: T]***					0.02	.88	300
Sure False	1	False: 7	4	False: 7			
Unsure False	6		3				
Unsure True	58	True: 93	20	True: 93			
Sure True	35		73				
***Item 10: A social marketing program for patients is likely to be more effective in a larger health system such as JerseyCare, as opposed to a smaller practice such as Johnson Family Practice. [Correct: F]***					0.15	.70	4
Sure True	16	True: 54	35	True: 56			
Unsure True	38		21				
Unsure False	34	False: 46	18	False: 44			
Sure False	12		26				
***Item 11: Tailoring aspects of the naloxone coprescription checklist to accommodate your practice is not recommended because it will limit the effectiveness of the naloxone coprescription program. [Correct: F]***					0.38	.54	−6
Sure True	7	True: 35	21	True: 33			
Unsure True	27		12				
Unsure False	41	False: 65	17	False: 67			
Sure False	24		50				
***Item 12: The RIOSORD tool calculates a patient’s risk of overdose according to his or her mental health comorbidities. [Correct: F]***					8.56	.003	117
Sure True	29	True: 87	63	True: 80			
Unsure True	58		17				
Unsure False	9	False: 13	5	False: 20			
Sure False	4		15				
***Item 13: Developing a stakeholder analysis can be an effective way to both engage and motivate stakeholders as well as facilitate buy-in to your coprescribing program. [Correct: T]***					18.67	<.001	−33
Sure False	3	False: 12	2	False: 4			
Unsure False	10		2				
Unsure True	56	True: 88	17	False: 96			
Sure True	32		79				
***Item 14: Organizational Readiness Assessments allow facilitators to identify the likelihood that instituting a change in their practice will be successful. [Correct: T]***					9.14	.003	−50
Sure False	2	False: 7	1	False: 3			
Unsure False	6		2				
Unsure True	57	True: 93	17	True: 98			
Sure True	36		81				
***Item 15: Gap analyses reveal unmet gaps in naloxone coprescribing to patients with a RIOSORD score of >18. [Correct: F]***					4.41	.04	191
Sure True	22	True: 84	64	True: 90			
Unsure True	63		26				
Unsure False	13	False: 16	6	False: 10			
Sure False	3		4				
***Item 16: Studies show that patients prescribed naloxone are more likely to engage in risky opioid-related behaviors because of a decreased perception of risk. [Correct: F]***					6.86	.009	45
Sure True	11	True: 34	16	True: 27			
Unsure True	23		11				
Unsure False	35	False: 66	12	False: 73			
Sure False	31		61				
***Item 17: Provider stigma is a barrier to coprescribing naloxone. [Correct: T]***					14.29	<.001	50
Sure False	2	False: 14	3	False: 5			
Unsure False	12		2				
Unsure True	29	True: 86	9	True: 96			
Sure True	57		87				
***Item 18: The RE-AIM^b^ framework is useful in structuring the evaluation and sustainability of your naloxone coprescription program. [Correct: T]***					10.12	.002	0
Sure False	1	False: 9	1	False: 3			
Unsure False	8		2				
Unsure True	58	True: 91	21	True: 97			
Sure True	32		76				
***Item 19: Providers in private practice with <10 staff members can implement the RE-AIM framework and naloxone coprescribing checklist effectively. [Correct: T]***					2.06	.15	67
Sure False	3	False: 16	5	False: 12			
Unsure False	14		7				
Unsure True	58	True: 84	26	True: 89			
Sure True	26		63				
***Item 20: In order to have the best results, implementation frameworks must be used in full and should not be combined. [Correct: F]***					6.88	.009	73
Sure True	22	True: 64	38	True: 57			
Unsure True	42		19				
Unsure False	28	False: 36	19	False: 44			
Sure False	8		25				

a RIOSORD: Risk Index for Overdose or Serious Opioid-Induced Respiratory Depression.

b RE-AIM: reach, effectiveness, adoption, implementation, and maintenance.

## Discussion

In summary, findings suggest that the naloxone coprescription academic detailing intervention was effective at delivering content area knowledge and stimulating metacognition about coprescription practices. From a knowledge gain perspective, the intervention saw increases in participant knowledge along the key objectives of a naloxone coprescription program. In addition, metacognitively, results suggest that individuals were more likely to be confident in their answer choices after the intervention. While the confidence gain was seen mostly among participants who chose correct answers, a small number of participants also became overconfident in their incorrect answers. This finding could support the development of refresher courses as a tactic to reexpose those who were overconfident to the material to correct any misunderstanding of course content [15], and for naloxone-prescribing programs, refreshers would be needed to account for the changing nature of the naloxone marketplace or clinical guidelines for overdose risk. The absolute AC significantly improved after intervention. Participants were better able to confidently discern correct and incorrect answers at posttest.

Across medical education settings, metacognitive evaluations have been implemented successfully, which has resulted in improvements in metacognition itself [16], the learning and retrieval of basic science information [17], and moderation of performance test anxiety in observed clinical examinations [18]. Even withstanding the complexity of metacognitive measurement concepts [10], CTF presents itself as a simple mechanism for metacognitive evaluation available to medical educators and evaluators, allowing them to assess potential areas of weakness in content delivery and specific areas where students may struggle with concepts [14]. Academic detailing programs applying metacognitive evaluative processes may be best served by developing feedback loops for learners and curriculum designers driven by the results CTF tests. In our results, learners were the most confident in incorrect answers for questions detailing the specifics of assessing individual risk of overdose. Feedback to learners could provide clarification on application of the RIOSORD tool through follow-up emails, refresher courses, or the development of learning communities to support implementation and adoption. Feedback to curriculum designers may prompt an evaluation of course content to identify what course updates were needed to ensure key concept delivery.

This specific intervention was self-paced and web-based, a common format available for CME. Electronic interventions have been shown to be no different for metacognition than in-person interventions, despite having no formal educator to guide the process [19]. This is important evidence to bolster the benefit of web-based continuing education [9], especially given the proliferation of web-based education that occurred during the COVID-19 pandemic [20]. Evidence suggests that if learners are going to engage in a self-paced curriculum, adding a metacognitive layer forces learners to critically think about their content knowledge acquisition [21]. The identified potential overconfidence observed in this study after receiving education is consistent with other metacognitive evaluations [22].

This study is not without limitations. The evaluation used a 1-group pretest, posttest design, which limits generalizability of the findings. While the course on best practices for coprescribing was brief and designed to be completed in 1 session, it is unknown how or whether other naloxone initiatives may have influenced participants. The academic detailing program’s enrollment was open to RUSOM and its affiliates; it is not possible to rule out selection bias as 1 factor influencing score improvements.

While metacognitive processing was shown to be important for behavior change, we do not have a long-term measure to determine whether the intervention resulted in increased naloxone prescription or even whether learners went on to implement coprescription initiatives in their practice settings. As referenced earlier, there are multiple ways to assess metacognition, of which CTF is one, and the validity of one accepted measure of metacognition has yet to be established. However, this particular method of assessing metacognition with multiple conceptual domains allows evaluators to use several diagnostic measures to understand the conditions under which knowledge gain is occurring in educational interventions. Future research could measure long-term changes in these particular scores, tracking metacognitive monitoring as skills are applied, and potentially correlate both cognitive and metacognitive changes with on-the-ground prescribing and implementation behaviors.

## Abbreviations

AC: accuracy of confidence judgments
BS: bias of confidence judgments
CCP: confidence correct probability
CIP: confidence incorrect probability
CME: continuing medical education
CTF: confidence-weighted true-false
DIS: discrimination between correct and incorrect decisions
RIOSORD: Risk Index for Overdose or Serious Opioid-Induced Respiratory Depression
RUSOM: Rowan University School of Osteopathic Medicine
